# Mono- versus polyaxial locking plates in distal femur fractures – a biomechanical comparison of the Non-Contact-Bridging- (NCB) and the PERILOC-plate

**DOI:** 10.1186/1471-2474-15-369

**Published:** 2014-11-06

**Authors:** Bilal Farouk El-Zayat, Turgay Efe, Steffen Ruchholtz, Salim Khatib, Nina Timmesfeld, Antonio Krüger, Ralph Zettl

**Affiliations:** Department of Orthopaedics and Rheumatology, University Hospital Marburg, Baldingerstrasse, 35043 Marburg, Germany; Department of Trauma, Hand and Reconstructive Surgery, University Hospital Marburg, Baldingerstrasse, 35043 Marburg, Germany; Institute for Medical Biometry and Epidemiology, Philipps University Marburg, Bunsenstraße 3, 35037 Marburg, Germany; Department of Orthopaedic Surgery, Kantonsspital Frauenfeld, Pfaffenholzstrasse 4, 8501 Frauenfeld, Switzerland

**Keywords:** Monoaxial and polyaxial locking plates, Biomechanical study, Distal femur fracture, NCB, PERILOC

## Abstract

**Background:**

The aim of this cadaveric study was to compare a polyaxial (NCB®, Zimmer) to a fixed-angle monoaxial locking plate (PERILOC®, Smith & Nephew) in comminuted fractures of the distal femur regarding stability of the construct. Up to date there is no published biomechanical data concerning polyaxial plating in cadaveric distal femurs.

**Methods:**

Fourteen formalin fixed femora were scanned by dual-energy x-ray absorptiometry. As fracture model an unstable supracondylar comminuted fracture was simulated. Fractures were pairwise randomly fixed either with a mono- (group A) or a polyaxial (group B) distal femur plate. The samples were tested in a servohydraulic mechanical testing system starting with an axial loading of 200 N following an increase of 200 N in every step with 500 cycles in every sequence up to a maximum of 2 000 N. The end points were implant failure or relevant loss of reduction. Data records included for each specimen time, number of cycles, axial load and axial displacement. Statistical analysis was performed using the exact Wilcoxon signed rank test.

**Results:**

The mean donor age at the time of death was 75 years. The bone mass density (BMD) of the femurs in both groups was comparable and showed no statistically significant differences. Five bones failed before reaching the maximum applied force of 2000 N. Distribution curves of all samples in both groups, showing the plastic deformation in relation to the axial force, showed no statistically significant differences.

**Conclusions:**

Operative stabilization of distal femur fractures can be successfully and equally well achieved using either a monoaxial or a polyaxial locking plate. Polyaxial screw fixation may have advantages if intramedullary implants are present.

**Electronic supplementary material:**

The online version of this article (doi:10.1186/1471-2474-15-369) contains supplementary material, which is available to authorized users.

## Background

Management of patients with articular distal femur fractures can often be difficult due to the complexity of the injury itself and to the often poor general condition of patients and their bones. Femur fractures are associated with complications that include malunion and implant failure[1]. Due to a relevant increase of elderly active patients with increased life expectancy and more hazardous sports more patients will present with femur fractures[2]. Two principle injury mechanisms are known: Either high energy trauma in younger patients e.g. after road traffic accidents or low energy trauma caused by falls in most elderly patients complicated through osteoporosis or even periprosthetic fractures[3]. The objectives in treatment include restoration of bone length, axis and rotation as well as a fixation that permits early full weight bearing to avoid immobilization and its associated complications[4].

As conservative treatment of distal femur fractures is obsolete, they used to be treated by (bicondylar) plate fixation in case of intact surrounding soft tissue or intramedullary nailing[5, 6]. However, the reported complication rate with non-union, loss of reduction and implant failure have been high[7–9]. Recent developments in the last decade described the principle of ‘minimal invasive *biological* plate osteosynthesis’ showing reduced blood loss, lower infection rates and possibility of bridging the supracondylar fracture zone as an internal fixator, preserving the bone fragments and the periosteum[1, 10–12]. The first and most famous of these products is the "Less Invasive Stabilizing System" (LISS®, Synthes Corp., Umkirch, Germany) showing improved biomechanical and clinical results[13]. Locking plates show advantages in osteoporotic bones for a lower rate of secondary dislocation as well as a definitely lower impairment of bone perfusion due to plate fixation with a small gap to the bone and the periosteum[3, 14]. The main downside of the monoaxial locking implants is that the existing thread in the plate pre-determines the perpendicular direction of the screw. That can often leads to positioning of screws in bone areas which intraoperatively have to be considered of minor quality. This may be associated with secondary loss of realignment and loosening of screws[15]. Ideas to avoid this and giving the surgeon more freedom in directing the screws through the bone lead to the introduction of polyaxial locking plates[16]. This implant (e.g. Non-Contact-Bridging-plate, Zimmer Inc., Winterthur, Switzerland) allows a locking screw placement in a range of 15° to the plate level. Additionally reduction of bony fragments (e.g. as lag screws) in the direction to the plate can be accomplished before the screws are locked. Angular stability is achieved by fixing the head of the screw with an additional cap turned into the plate thread covering the screw head (load to friction). Up to now, several clinical observations have been published concerning fixation of femur- and periprosthetic fractures with NCB plates[12, 16, 17] showing same or better results as compared to similar procedures with monoaxial implants. From the biomechanical point of view individual studies were published, comparing the available locking plates with synthetic bones only, but not on a cadaveric distal femur. As synthetic bone models will not capture interspecimen variability nor material inhomogeneity, both of which may affect relative performance of both implant systems, we performed the first cadaveric study to supply data that is closest to the real situation.

## Methods

### Specimens

Prior to receipt of donated bodies for teaching and/or scientific purposes at the University of Marburg, Department of Anatomy, consent is established using a Body Consent Form. Fourteen formalin fixed cadaveric femora were used. Conventional anteroposterior and mediolateral radiographs were taken from all specimens excluding preexisting pathology or prior fractures. Bone mineral density (BMD) was assessed using dual-energy x-ray absorptiometry (DXA, Lunar Prodigy, General Electric Company Healthcare, United Kingdom) imaging at the total hip. Left and right femora of one individual were randomized to either Group A (NCB) or Group B (PERILOC), allowing for a pair-wise comparison in an attempt to control for specimen variability. After truncating the femora to a length of 30 cm from the condyles, the proximal shaft was embedded in a special form cup with 8 screws in 90° opposition to each other. The femur condyles were positioned parallel to the horizontal axis. In this way, the weight bearing axis was oriented along the femur shaft axis. This research complies with the Declaration of Helsinki.

### Implants and locking mechanism

Both implants are from titanium alloy and anatomically preformed for left and right femurs to fit the lateral cortex. As a polyaxial locking plating system the 9-hole (246 mm) NCB® (Non-Contact-Bridging-plate, Zimmer Inc., Winterthur, Switzerland) was used[18]. Shaft fixation is achieved with 5 mm cortical screws, drilled with a 4.3 mm tip. For condyle fixation, 5 mm cancellous screws are used with a drill hole of 2.5 mm.

As reference implant, the monoaxial plating system with the 10-hole (230 mm) PERILOC plate (Smith & Nephew Inc., Cordova, USA) was used with a well known head locking construct. The plate was fixed to the shaft with 4.5 mm self-threading cortical screws with a 3.5 mm drill hole. The condyles were stabilized with 4.5 mm cancellous screws and a 3.5 mm drill hole.

### Fracture model

As a fracture model, an unstable supracondylar comminuted T-fracture was simulated (AO/OTA33-C2). A standardized osteotomy was performed vertically transcondylar in the "whiteside line" and horizontally in the supracondylar region. To mimic a comminuted fracture zone, a vertical gap of 20 mm was kept at plate fixation[19]. Correct fitting of the instrumentation was assessed and documented by x-ray as well as by photography for each specimen.

### Group A (NCB)

**Figure 1:**
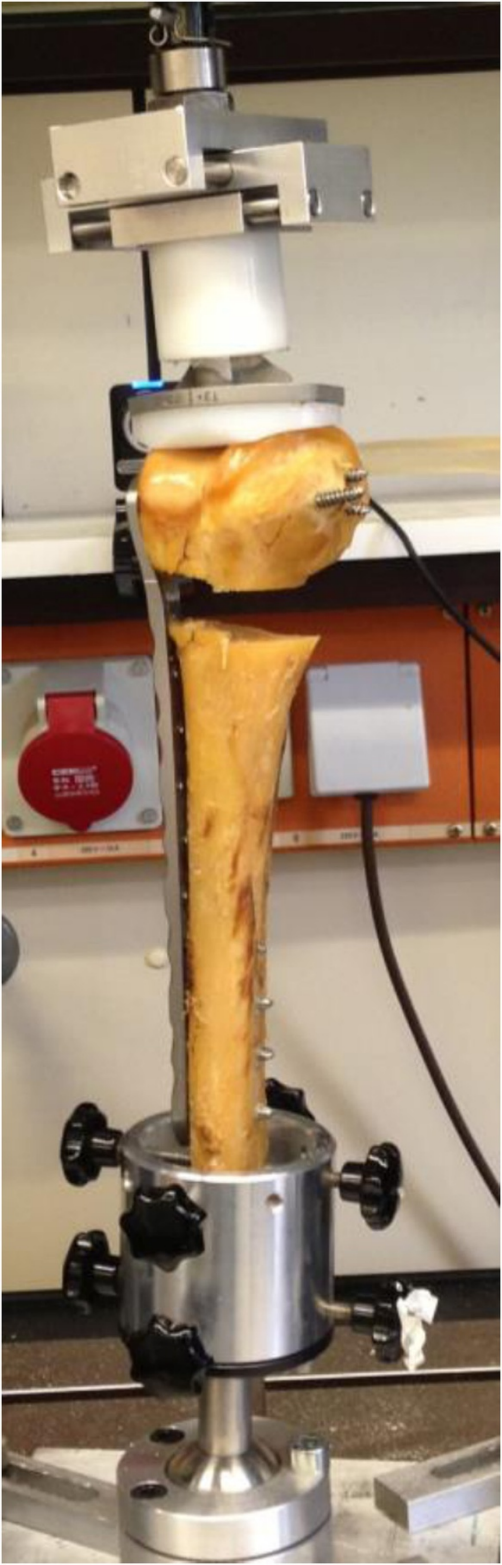
**Biomechanical setting.**

The NCB plate was fixed proximally with four 5 mm polyaxial locking screws in a 15° angle alternating in the direction to anterior and posterior. Distal to the comminuted segment all five plate holes were filled with 5 mm cancellous screws inserted at the maximum possible angle of 15° alternating for each screw. Following the manufacturer’s instructions locking caps were then applied to all screws using a torque-limiting screwdriver (6 N) provided with the system (Figure 1).

### Group B (PERILOC)

The PERILOC plate was fixed to the shaft with four 4.5 mm cortical screws perpendicular to the plate. For distal fragment fixation the same number of five 4.5 mm cancellous screws were inserted.

### Biomechanical testing

The biomechanical testing was performed on a servohydraulic mechanical testing system (series 5566, Instron Corporation, Norwood, MA, USA). For realistic simulation the femur was turned upside down. As a pressure device, a polyethylene covered tibial baseplate of a total knee arthroplasty was used and mounted on the testing machine proximally. The proximal femur shaft was fixed distally in a custom-made mold to prevent rotation during testing. At all times of testing, the proximally positioned tibial condyle was free to move under the load application plate.

Each specimen was tested under incrementally increasing cyclic sinusoidal loads applied vertically through the anatomical axis of the femur. We used a loading protocol well described for mechanical evaluation of distal femur fractures[19–21]. It consisted of increments of 10 cycles, starting with a 200 N load. The load of each following increment was increased by 200 N to a maximum load of 2000 N with 500 cycles at a frequency of 1 Hz allowing 15 minutes of rest between each increment. Testing was conducted in a displacement control mode.

### End points

The test end point was implant breakage or relevant loss of reduction due to cutout of the screws in the bone. Break off criteria for the biomechanical testing as construct failure were defined as a sudden load drop of more than 30 % or plastic deformation of more than 5 mm.

### Data acquisition and statistical analysis

Time, number of cycles, axial load and axial displacement were recorded using Bluehill II software, series 5500. For axial testing, a load–displacement curve was plotted for each construct. Plastic deformation was calculated by subtracting the initial displacement from displacement present after reaching yield point once the load was removed. It subsumes the deformation of the bone, the screws and the plate, whereas the weakest component of this chain is the cadaveric bone. Synchronously, optical (photo and video) and machine data were captured.

Statistical analysis was performed using the R program for statistical computing (http://www.r-project.org; version 2.15.0; package MethComp). Due to a low sample number a non parametric statistical test (exact Wilcoxon signed rank test) was applied. A *p*-value of less than 0.05 was considered to be statistically significant.

## Results

**Figure 2:**
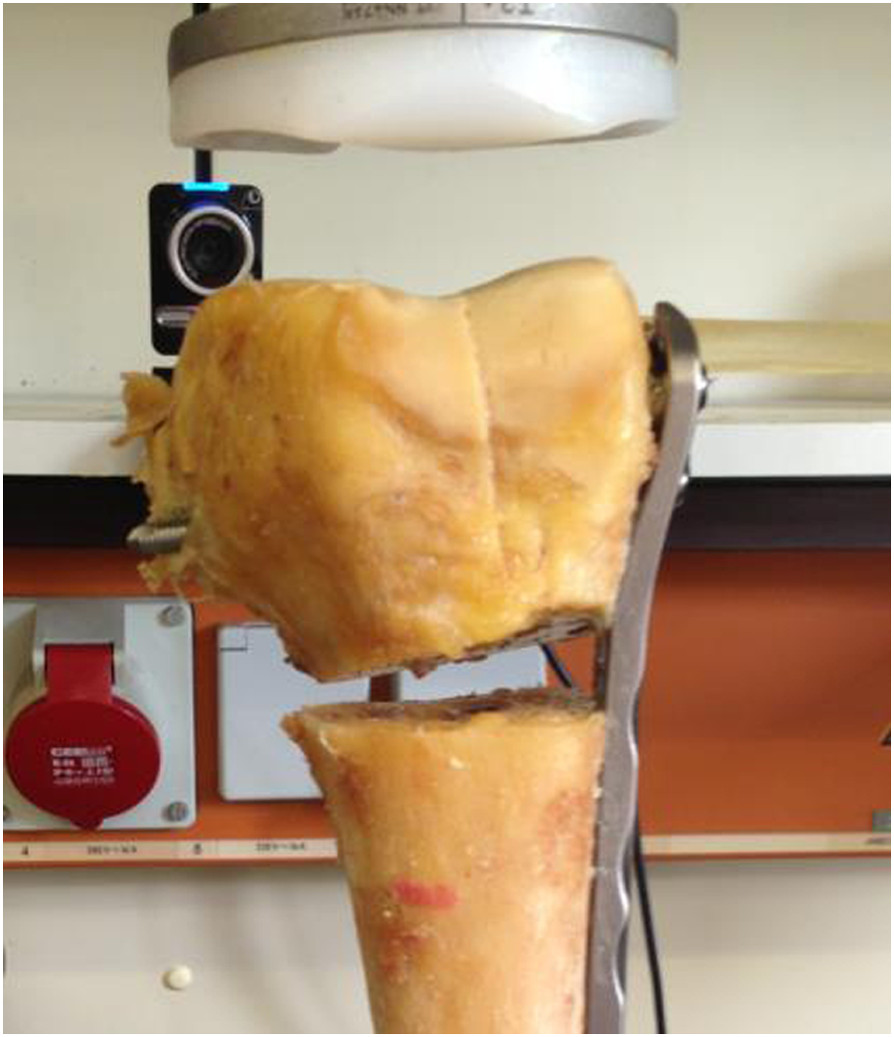
**Failure at 1600 N (NCB-plate, sample 11/10) with deformation of the bone due to a cutout of the screws.**

**Figure 3:**
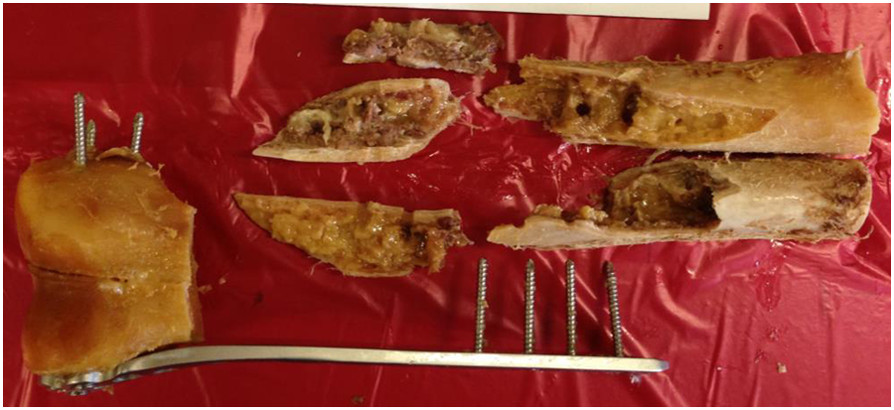
**Complete pull out of the proximal plate and fracture at 2000 N at an osteoporotic bone.** PERILOC®-plate (sample 17-10).

**Figure 4:**
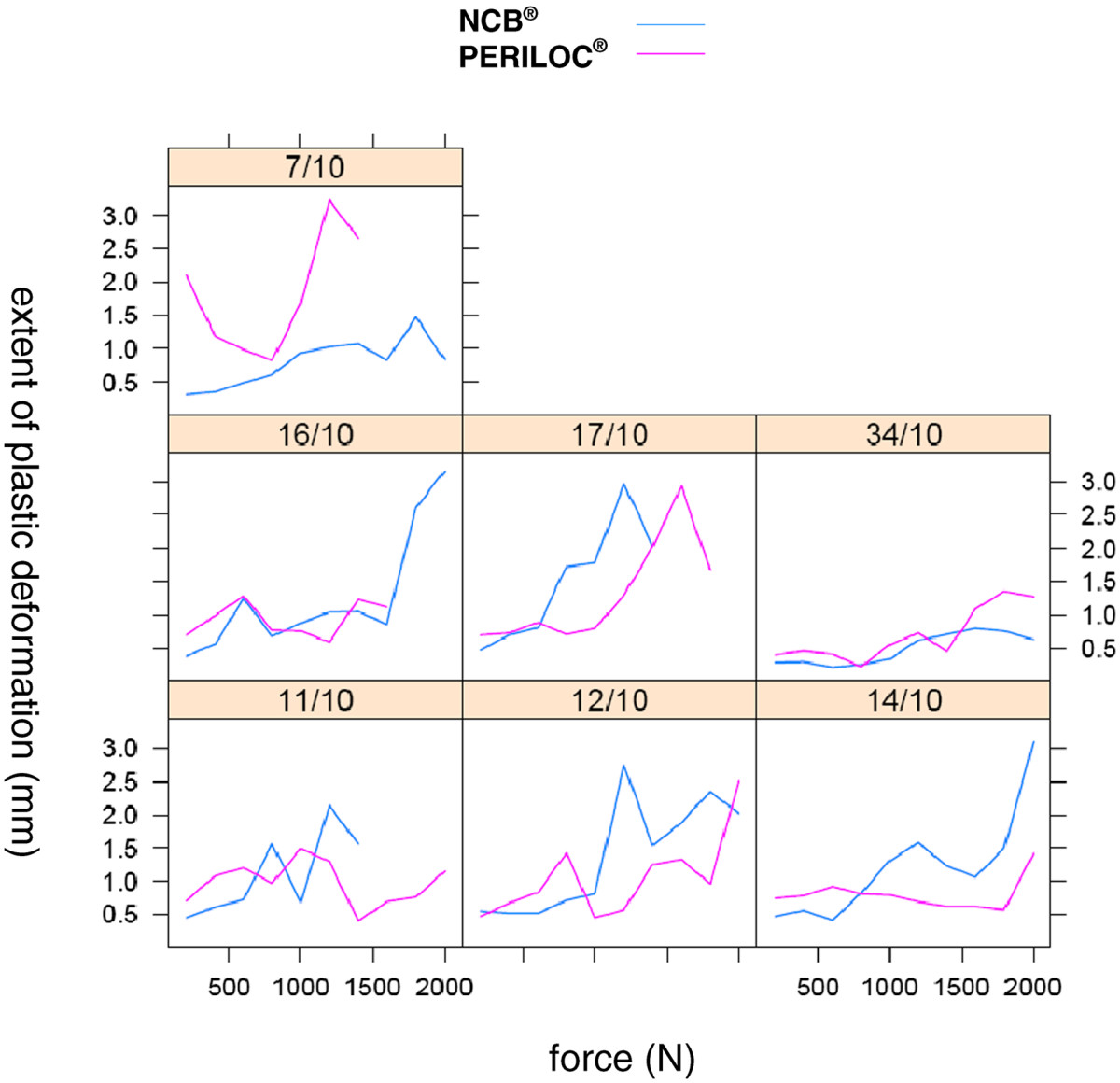
**Absolute values of plastic deformation of all probe pairs (the scales are for all graphs the same).**

**Figure 5:**
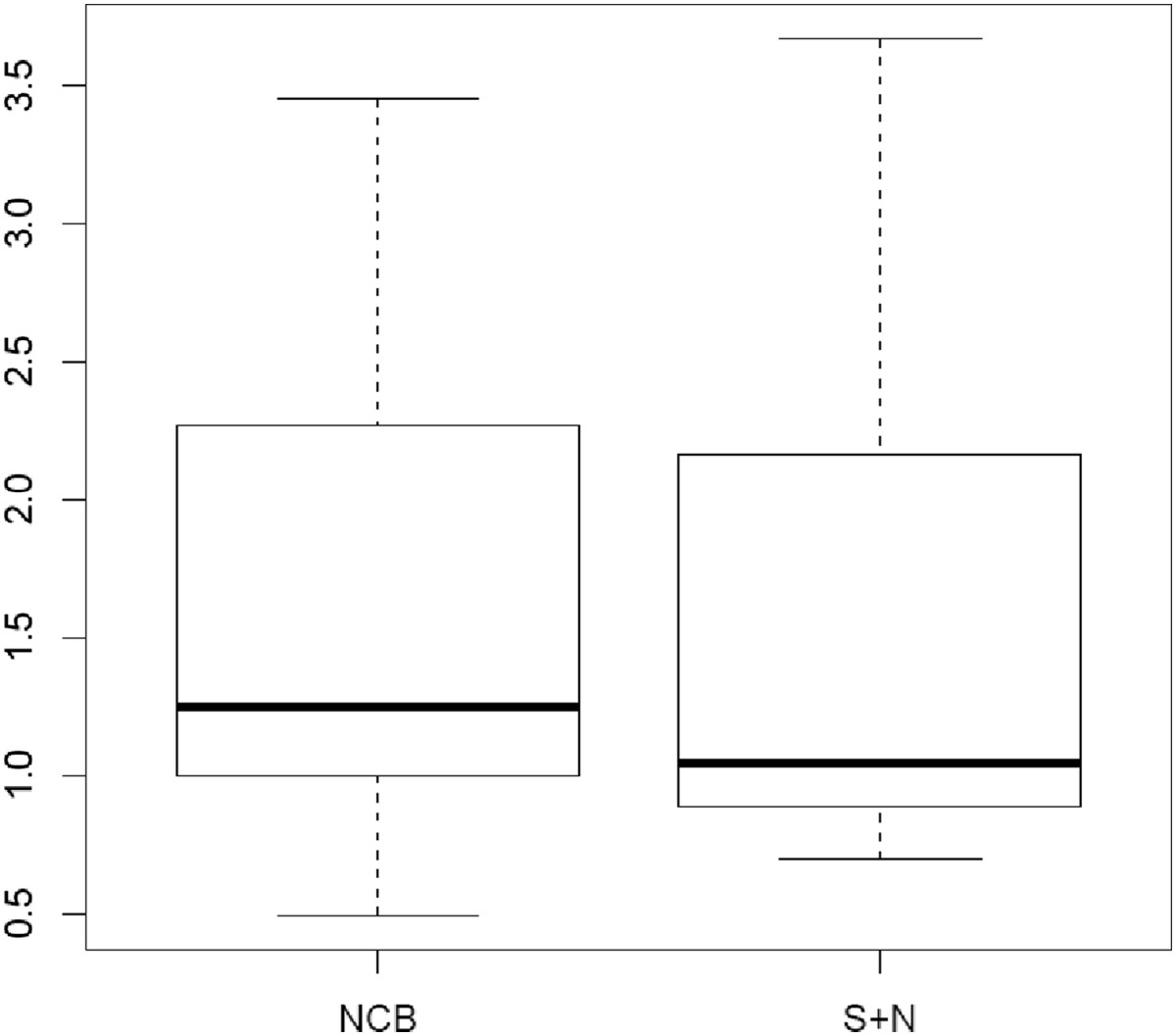
**Boxplots of the mean plastic deformation for each plate (in mm).** The box shows the area of the middle 50% of the data. The horizontal line in the box represents the median while the whiskers reach out to the minimum and maximum values.

Seven pairs of cadaveric femora were used (2 male, 5 female donors). The mean donor age at the time of death was 75 years (range 45-92 years). The results for the biomechanical testing are shown in Table 1.Five constructs failed before reaching the maximum applied force of 2000 N. In 4 cases, failure was due to a distal loss of reduction (2 × PERILOC, 2 × NCB) due to a cutout of the screws as shown in Figure 2.One sample (PERILOC®) showed at 2000 N a complete failure in the shaft region, whereas the condyles were still stable (Figure 3). All other bones reached the test endpoint of 2000 N without any complication.Figure 4 presents the absolute values of plastic deformation of all samples in relation to the axial force. Both plates are compared for every bone pair. In most samples the curves have a very parallel or similar course.The overall plastic deformation in both groups was comparable. The measured differences were statistically not significant (Figure 5).

**Table 1:** **Comparison of bone mineral density and plastic deformation of each construct [data is given as mean value ± standard deviation (SD)]**

	Group A (NCB®)	Group B (PERILOC®)	p-value
	n =7	n =7	
Bone mineral density (g/cm^2^)	0.799 ± 0.226	0.855 g/cm^2^ ± 0.263	*p* = 0.699
	Range 0.423 – 1.126	Range 0.555 – 1.313	
Plastic deformation (mm)	1.51 ± 0.77	1.37 ± 0.83	*p* = 0.717
	(Range 0.81 – 3.26)	(Range 0.83 – 3.67)

In the radiographs, we observed in all specimens of both groups no unexpected results (bone fissures or fractures, screw loosening/loss of locking mechanism, screw bending) despite the 5 described construct failures. No obvious permanent plate bending or deformation was found. Only one pullout failure of the proximal fragment as a complete burst fracture was observed as shown above.

## Discussion

Distal femur fractures especially in the elderly are dedicated to be the most problematic fractures in trauma surgery. These fractures can be complicated to treat and require advanced surgical skills and optimal implants. After nailing and conventional plating, monoaxial plating was suggested[9, 17, 22]. The newest device is the polyaxial locking plate.

From a clinical point of view, there are two main targets: first, that the fracture heals, and second, that the construct itself does not fail[8]. Stress tests have indicated that the locking plates of the first generation may provide improved distal fixation of distal femur fractures especially in osteoporotic bone, withstanding greater axial loads and requiring higher energy to failure, compared to the angled blade plates or intramedullary nails[23, 24]. In far distal fractures sufficient fixation of an intramedullary nail can be impossible, why in such cases plating should be preferred[4]. To maintain the osseous blood supply, pre-contoured plates are placed in an epiperiosteal position, and should have no direct contact with the bone. Several clinical and biomechanical studies evaluated such a system (LISS® - Less-Invasive stabilization System) which was introduced as an "internal fixator"[25]. However, this and other first-generation fixed-angle locking plates have screws that are inserted perpendicular to the plate, which limits their clinical applicability[4, 5, 20, 26–28]. In the case of periprosthetic fractures with intramedullary stems or impossible bicortical screw placement, unicortical screw fixation was suggested. Unfortunately, several studies show high complication rates of up to 30% due to a break-out of the unicortical screws especially in osteoporotic bones[29–31]. Polyaxial screw placement is suggested to be the clue.

Up to date only two authors published biomechanical series comparing the polyaxial NCB®-plate to monoaxial implants in the distal femur. Otto et al. compared the stability to axial loading to the LISS® plate in synthetic bones. There was no difference with regard to stiffness, load to failure, and peak force[32]. Wilkens et al. compared the NCB®-plate to another polyaxial plate and the LISS® furthermore in terms of parallel or angled screw placement[21]. They showed that the NCB® system outperformed the conventional plates in nearly all tested parameters. As limitation of their study Wilkens et al. stated that it was not conducted with cadaveric bones[33]. Investigations using synthetic bones can not adequately replace cadaveric studies due to the fact that synthetic bone models will not capture interspecimen variability nor material inhomogeneity, both of which may affect relative performance of both implant systems. Nevertheless these studies may need to be validated by further testing with a small cadaveric bone sample in order to produce conclusive results. The only two publications delivering biomechanical data with cadaveric femurs and NCB® plates are dealing with proximal periprosthetic fractures. Konstantinidis et al. compared bi- (NCB®)- and monocortical LISS®- fixation in formalin-fixed femora with Vancouver B1-fractures[34]. They concluded that bicortical screw placement should be preferred whenever possible and that fracture stabilization can be achieved equally well using either LISS®- or the NCB®-plate. Wähnert et al. published recently a biomechanical comparison with fresh-frozen cadavers of the same periprosthetic proximal femur fracture. They compared the NCB® system to another angular stable implant (fixed angle locking attachment plate, LAP®, DepuySynthes) and concluded that the non-contact bridging plate revealed significantly higher failure load and may be the preferred option[35].

There is no published data yet comparing NCB® plates to monoaxial plates in a *cadaveric* model of a distal femur fracture. The PERILOC® plate was chosen as monoaxial reference as it is from the biomechanical aspects as well as from the locking mechanism (thread in the head of the screw engaging in the hole of the plate) comparable to the LISS® plate. Both system simulates the concept of minimally invasive bridging of a comminuted fracture, and could be implanted via an aiming device with a limited approach. Concerning different available cadaveric bones a prior study showed, that formalin-fixed and fresh frozen femurs had similar characteristics in mechanical testing[36]. In the present study cadaveric formalin fixed femurs were used to mimic a realistic situation in such fractures in either osteoporotic as well as in younger individuals with higher bone density. The BMD in both groups showed samples either from osteoporotic as well as from younger and stronger bones. Both groups showed in regard of BMD no significant differences. The study design aimed at testing plate osteosynthesis at an open gap fracture model, which was biomechanically similar to a comminuted, inaccurately reduced, or non-consolidated fracture. A major challenge in real surgery is the direction of screws from a weak or comminuted region to a subjective ‘more stable’ bony region in internal fixation. Fixed angle monoaxial plates do not allow any angulation of the drill for screw placement, leading to a frequent suboptimal screw fixation in weak cancellous bone. Polyaxiality allows the surgeon to redirect the drill to find subjective ‘stronger’ bone for securer screw placement in the condylar region, as well as in the shaft (use of lag screws as a kind of hybrid system).

Concerning the locking mechanism of both systems, the above mentioned results showed no failure of locking mechanism or screw loosening. This is comparable to the published biomechanical data showing sufficient stability of the innovative frictional locking mechanism of the NCB® system with small caps locking the screw heads to the plate[21].

In our series, no implant failure as plate breakage or bending occurred. This may be due to the limited force applied on the samples with 2000 N and to the "physiological" loading with the axial force on the anatomical axis. There was no scientific interest in maximal loading like a "load-to failure" as the results up to a relevant high force were meaningful. Comparing both devices in both groups, no statistically significant differences were found regarding stability. The failure modes, which were observed in other biomechanical series with similar test designs at the femur shaft or proximal femur are analog to our results[37].

This study, as in several other clinical and composite bone trials[30–32, 34], shows at least similar stability of tested poly- and monoaxial plates.

This study is limited by its inability to directly correlate biomechanical testing in a cadaveric system with clinical results. Another limitation results from using formalin-fixed specimen denuded of surrounding soft tissue. However, as shown by Topp et al. they have similar characteristics in mechanical testing as fresh frozen bones and are a good option for mechanical testing of orthopedic and trauma devices[36]. Moreover, the use of paired femora will likely limit any confounding effect of the formalin-related alterations of biomechanical properties of the specimen.

## Conclusion

The test settings used in this study showed no statistical difference in the stability of PERILOC® and NCB® plates. No material fatigue under cyclical loading was observed. Thus, the NCB®-plate is comparable to the PERILOC®-plate in biomechanical aspects. Operative stabilization of distal femur fractures can be successfully and equally well achieved using either the PERILOC® plate or the NCB® plate. In complex supracondylar and periprosthetic fractures, the NCB® -plate might show superiority due to its higher flexibility and angulation in bicortical screw placement to regions with better bone stock.

## Authors’ original submitted files for images

Below are the links to the authors’ original submitted files for images. Authors’ original file for figure 1 Authors’ original file for figure 2 Authors’ original file for figure 3 Authors’ original file for figure 4 Authors’ original file for figure 5
