# Supplementary figures and images for: Mono- versus polyaxial locking plates in distal femur fractures – a biomechanical comparison of the Non-Contact-Bridging- (NCB) and the PERILOC-plate
Source: BMC Musculoskelet Disord. 2014 Nov 6;15:369. doi: 10.1186/1471-2474-15-369 (PMC4232626; doi:10.1186/1471-2474-15-369)

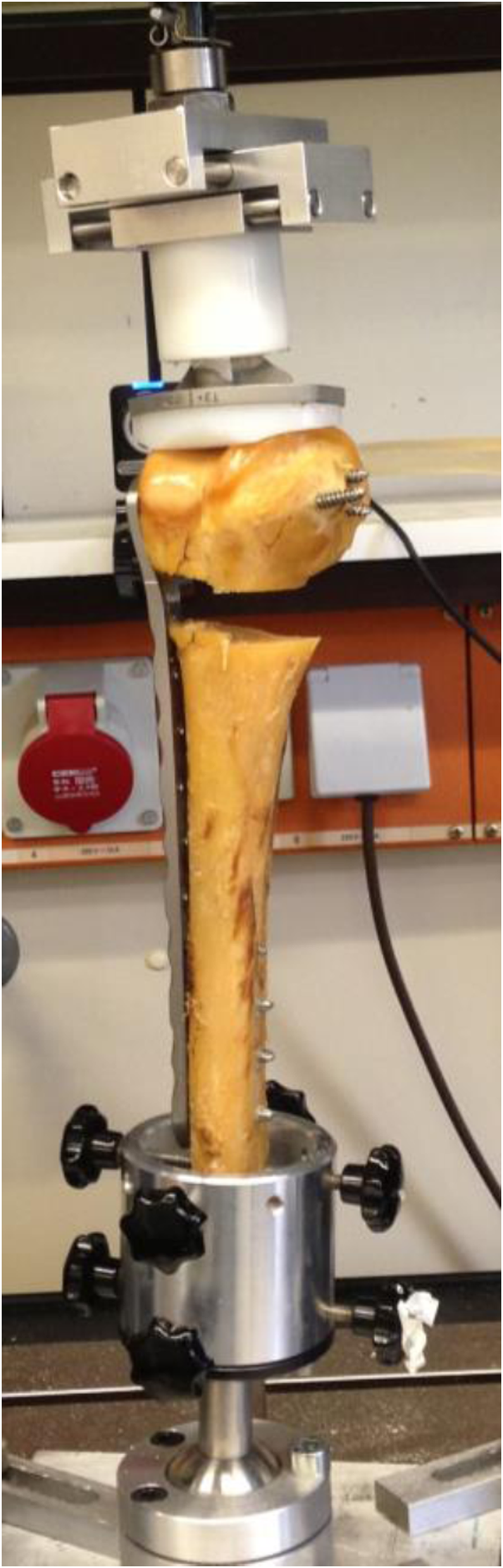

Supplement: Supplementary file 1 — Authors’ original file for figure 1 [file 12891_2014_2305_MOESM1_ESM.tif]

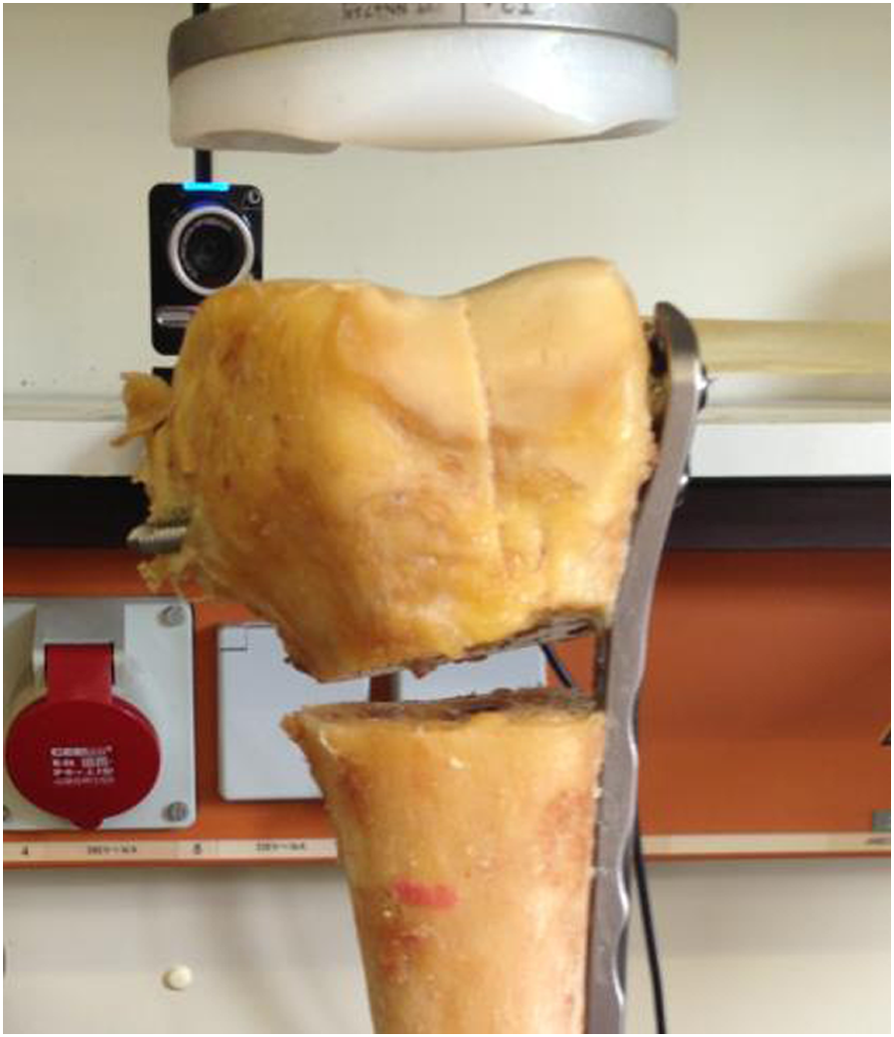

Supplement: Supplementary file 2 — Authors’ original file for figure 2 [file 12891_2014_2305_MOESM2_ESM.tif]

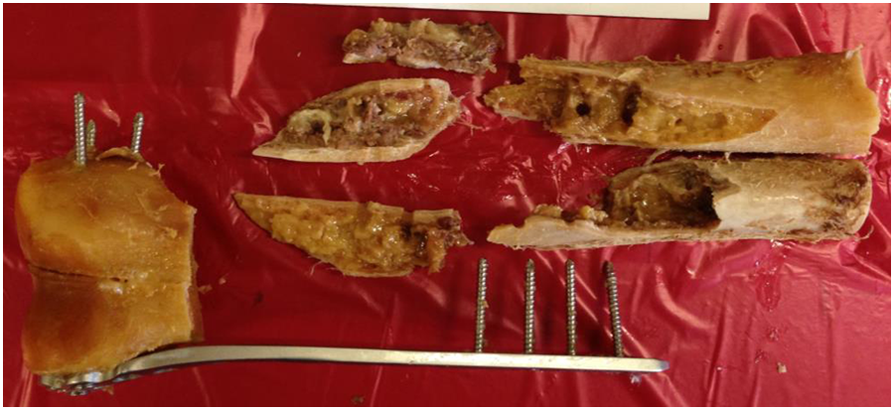

Supplement: Supplementary file 3 — Authors’ original file for figure 3 [file 12891_2014_2305_MOESM3_ESM.tif]

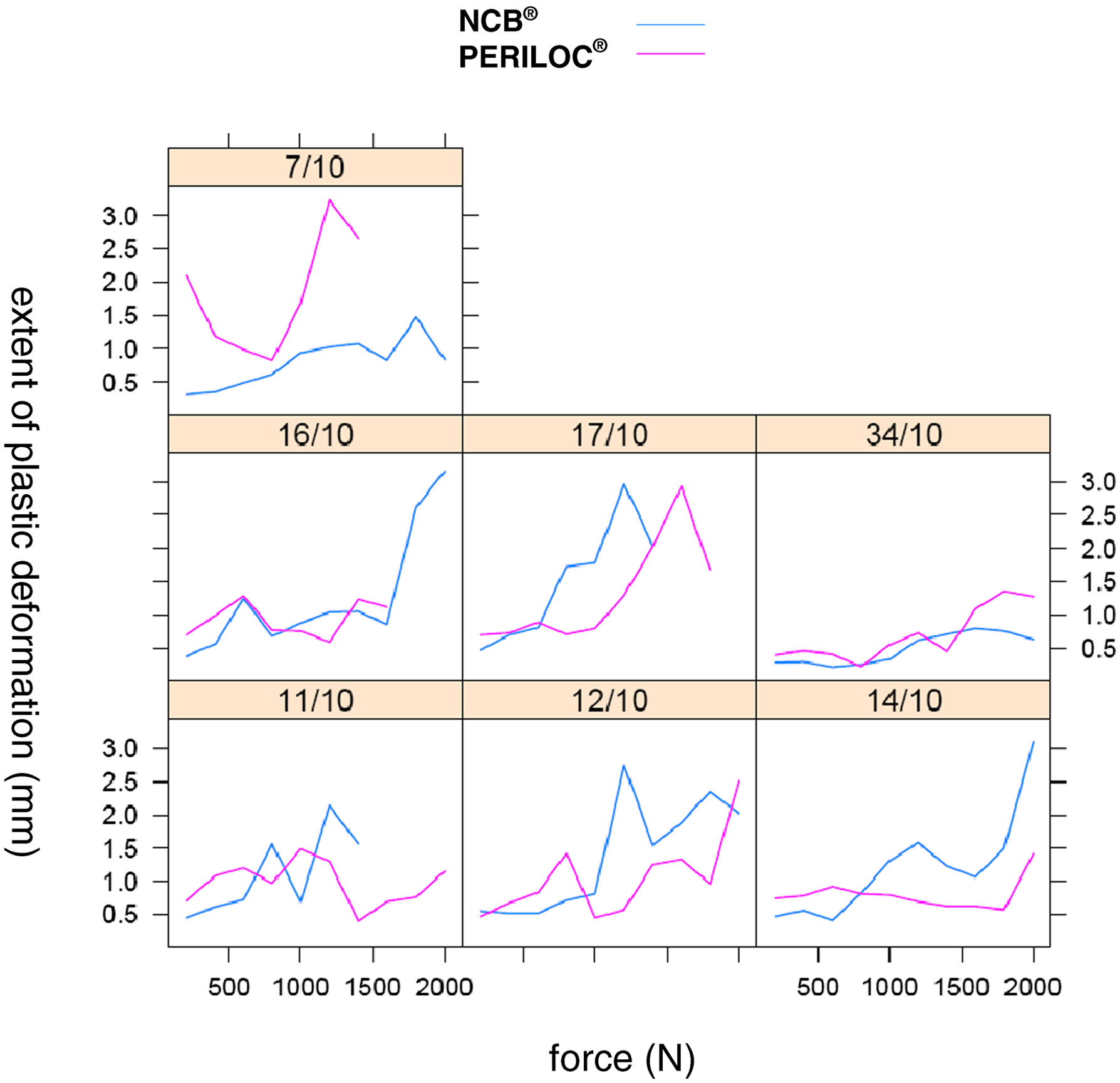

Supplement: Supplementary file 4 — Authors’ original file for figure 4 [file 12891_2014_2305_MOESM4_ESM.tif]

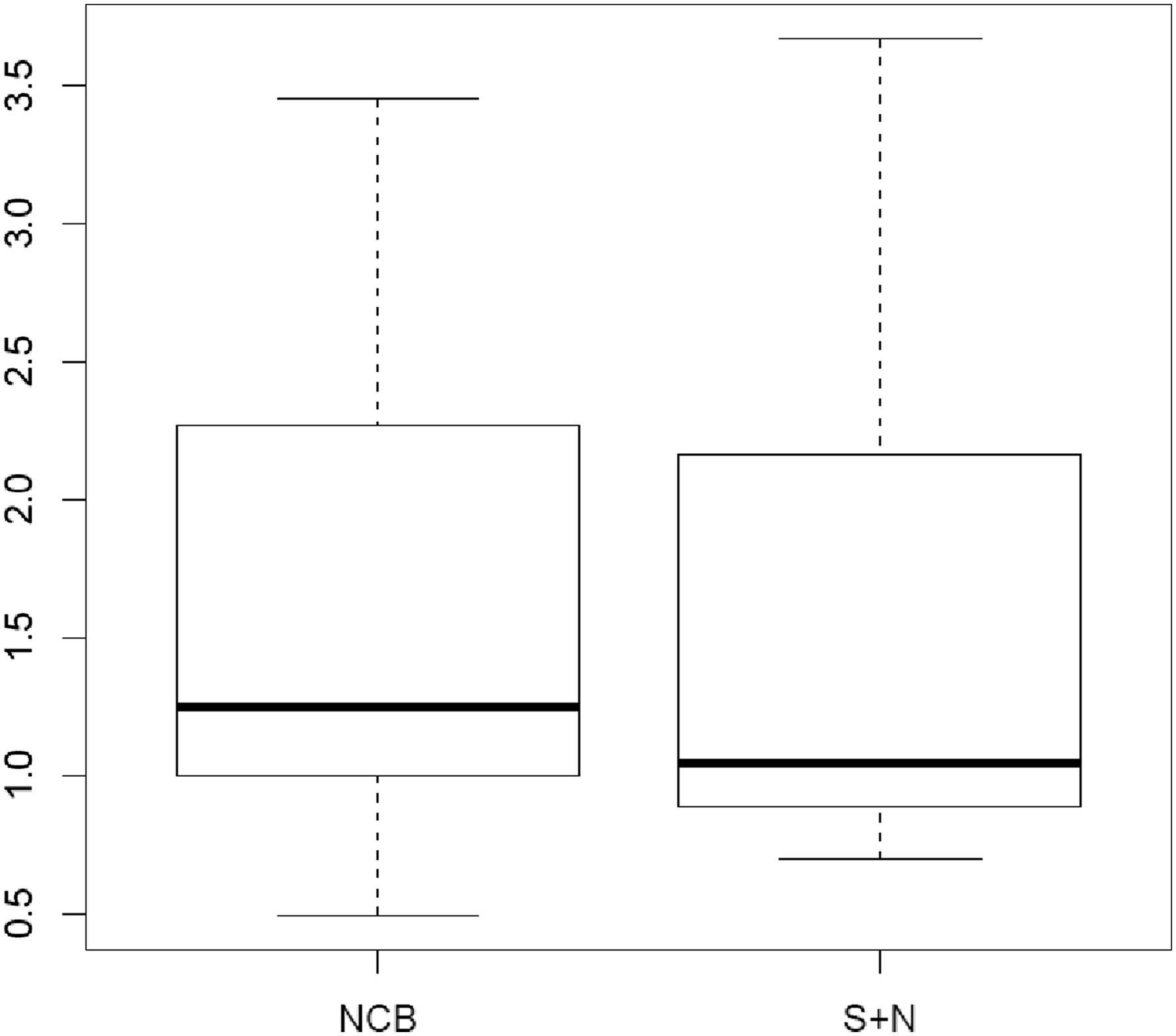

Supplement: Supplementary file 5 — Authors’ original file for figure 5 [file 12891_2014_2305_MOESM5_ESM.tif]
